# Accuracy of the My Jump Lab App for Two Methods of Single-Leg Countermovement Jump Height Assessment

**DOI:** 10.3390/jcm15187052

**Published:** 2026-09-11

**Authors:** Jarosław Kabaciński, Michał Murawa

**Affiliations:** Department of Biomechanics, Poznan University of Physical Education, 61-871 Poznan, Poland; murawa@awf.poznan.pl

**Keywords:** validity, My Jump Lab app, single-leg countermovement jump, flight time, sports medicine, biomechanics

## Abstract

**Background/Objectives**: The My Jump Lab app can be used to examine inter-limb asymmetry during the countermovement jump (CMJ), both in healthy athletes and in athletes undergoing rehabilitation post-injury. However, the correct assessment of jump height (JH) using this application requires high measurement accuracy, similar to the method of double integration of vertical ground reaction force values. This study aimed to determine the validity and reliability of the My Jump Lab app for estimating jump height (JH) during the single-leg countermovement jump (CMJ). **Methods**: Twenty-two healthy male adults performed single-leg CMJs for the dominant lower extremity (D) and non-dominant lower extremity (ND). The AMTI force platform and an iPhone 13 smartphone were used. JH during the CMJ was estimated based on the displacement of the jumper’s center of mass (force platform), the jumper’s flight time (smartphone and My Jump Lab), and the flight time of the reflective marker placed on the jumper’s sacrum (smartphone and My Jump Lab-M). **Results**: The assessment of the concurrent validity showed (1) poor agreement between the My Jump Lab and the force platform for the ND and D (*p* < 0.001) and (2) moderate (ND) and good (D) agreement between the My Jump Lab-M and the force platform (*p* < 0.001). **Conclusions**: The results of the single-leg CMJ height estimation revealed the greater accuracy of the My Jump Lab-M method compared with the My Jump Lab method. However, due to the good agreement between My Jump Lab-M and the gold standard, this new method may not provide an objective assessment of the height in this vertical jump. Therefore, the most accurate method of double integration of vertical ground reaction force values is recommended for the single-leg CMJ test.

## 1. Introduction

The countermovement jump (CMJ) is usually used to test jumping ability, power and explosive strength. Assessing these variables during the CMJ provides important data about the athlete’s physical capabilities in the context of improving athletic performance and preventing injuries [1,2]. Furthermore, the single-leg CMJ has gained wide utility in examining asymmetry between limbs, both in healthy athletes and in athletes undergoing rehabilitation [3,4,5,6]. The results of the single-leg CMJ in athletes often indicate increased inter-limb jump asymmetry (above 10% or 15%), which may impair exercise performance, negatively impact sports results [7,8,9,10,11], and result in reduced performance in the weaker limb and increased risk of injury [6,12,13,14].

Vertical jump height (JH) is a key biomechanical parameter, extremely useful in assessing an athlete’s neuromuscular performance and identifying injury risk factors [1,2]. JH, as the maximum displacement of the jumper’s center of mass (CM) during the flight phase, is commonly determined by the accurate method of double integration of the vertical ground reaction force (VGRF) values, as recorded by a force platform. Furthermore, vertical JH can be estimated by measuring flight time using the Bosco formula [15]. In addition to contact mats or the Optojump system, the assessment of JH from flight time can also be provided by smartphone applications, especially those installed on an iPhone equipped with a high-resolution camera. After recording the jump with a smartphone camera, the app analyzes the video to estimate, among other things, JH based on flight time, calculated by marking the take-off and landing moments on a timeline [16].

The My Jump app (both version 2 and Lab) has gained popularity recently, is much cheaper and more practical than laboratory devices, and does not require complicated configuration [16,17,18,19,20,21,22]. Therefore, this alternative tool can facilitate diagnostics performed by practitioners, including physiotherapists, when monitoring the progress of patients’ rehabilitation, e.g., after different knee joint injuries. Regularly recording vertical jumps, such as the CMJ, using this app helps identify asymmetries and sudden declines in performance, which protects athletes from the recurrence of the injury. Additionally, the My Jump Lab app includes an AI feature that enhances these analyses by using computer vision (image recognition) to create a box around the object captured in the video and calculate its pixel position during the jump [21,23].

Validation of this smartphone app has commonly included a comparison of this tool with a force platform as the criterion (gold standard) for assessing vertical JH [16,17,18,19,20,21,23,24,25,26,27,28,29,30,31,32,33,34,35]. Previous studies have reported very high agreement between My Jump and a force platform for CMJ height calculated based on flight time [16,21,23,24,25,26,27,29,30,31,32,33,34]. Furthermore, using the impulse–momentum method, some researchers have demonstrated similar CMJ height values and very high intraclass correlation coefficients between these measurement tools [23,24,36]. Considering the most accurate method, Murawa et al. [20] validated the My Jump 2 app by comparing it with a force platform for JH obtained using a jumper’s CM trajectory after double integration of the VGRF values. These authors established poor agreement between My Jump 2 and this method for recording the double-leg CMJ. In the case of single-leg jumps, Stojiljković et al. [32] revealed the high accuracy of My Jump 2 (ICC above 0.9) for JH and the asymmetry index, albeit using flight time in subjects performing the drop jump.

A review of the available literature showed that previous studies have not validated the My Jump Lab app for single-leg CMJ height calculated using the double integration of VGRF values. The present study was designed to evaluate the accuracy of the My Jump Lab app for two flight time methods compared to the most accurate method as the criterion. Therefore, the aim of this study was to determine the concurrent validity and absolute reliability of the My Jump Lab app for estimating JH using the jumper’s flight time (standard method) and the flight time of a reflective marker placed on the jumper’s sacrum (new method) during the single-leg CMJ.

## 2. Materials and Methods

### 2.1. Participants

Participants included twenty-two healthy and recreationally active male students from Poznan University of Physical Education (age: 20.8 ± 1.7 years and range: 18–23 years; body mass: 73.9 ± 11.5 kg; and body height: 1.79 ± 0.08 m). An a priori power analysis was conducted using G*Power (version 3.1.9.7). We assumed an effect size of 0.3 (the value between a medium effect and a large effect), a power of 0.80, an alpha level of 0.05, 1 group, 3 measurements, 0.5 correlations among repeated measures and non-sphericity, 1 correction, and a total sample of 20. The International Physical Activity Questionnaire (IPAQ) was used to determine the physical activity of the participants [37]. According to the IPAQ guidelines, students obtained a high activity level (18 participants) and a sufficient activity level (4 participants). In addition, each student declared the right lower extremity (LE) as functionally dominant during various movements (e.g., jumping, kicking). This dominance was confirmed in all participants by a ball-kicking test performed in the laboratory. Hence, the dominant LE (D) and the non-dominant LE (ND) were adopted. The inclusion criteria included participants (1) aged between 18 and 24 years; (2) with no professional sports practice; (3) with no history of ankle, knee, hip, or back injuries (one year before testing); (4) with no potential medical problems; and (5) with at least a sufficient level of physical activity based on the IPAQ. The study was conducted in accordance with the Declaration of Helsinki and was approved by the Ethical Committee of the Poznan University of Medical Sciences (number 546/16, 10 June 2022). Informed written consent was obtained from all participants.

### 2.2. Data Collection

The AMTI BP400600 force platform, with a sampling rate of 800 Hz (Watertown, MA, USA), was used. This platform features a 4th-order zero-phase, low-pass Butterworth filter with a cut-off frequency of 30 Hz to 50 Hz. The GRF values were collected using the BTS Smart Capture software (version Smart-D, BTS Bioengineering, Milan, Italy). The maximum jump height (JH) for the dominant LE (D) and non-dominant LE (ND) was assessed during the single-leg countermovement jump (CMJ). These tests were conducted over two days from 10 a.m. to 3 p.m., i.e., on the first day, 11 participants were tested, and on the next day, 11 participants were also tested.

All participants were familiarized with the experimental procedures. Measurements were preceded by a 10 min warm-up involving treadmill running (2 min), as well as isometric exercises (2 min) and dynamic stretching exercises (6 min), such as leg swings, skips, squats and light jumps. After a few trials, the jumper performed three CMJs for D and three CMJs for ND, in any order (D or ND) and alternately (first one LE and then the opposite LE). These six single-leg CMJs were considered successful if they were performed according to established guidelines during trial jumps and recorded by measurement tools. Otherwise, the participant repeated the incorrect jump. The jumper started the CMJ from an upright position, standing on one LE and keeping the opposite LE raised above the ankle. In the standing phase, each participant was required to maintain a stable body position for approximately 2 s (the “stand still” command). After the “start” command, the jumper flexed their knee joint to an angle of approximately 90° (eccentric phase) between the longitudinal axis of the lower leg and the longitudinal axis of the thigh. Then, the jumper immediately extended their knee joint and took off from one foot (concentric phase). Furthermore, all participants were instructed to squat to 90° at the knee joint, keep their hands on their hips, jump as high as possible, and land on the same foot during the CMJ. The rest period between jumps was approximately half a minute.

The My Jump Lab app, installed on an iPhone 13 smartphone (Apple Inc., Cupertino, CA, USA) with iOS 17, was used for testing. All CMJs were recorded using the camera of this smartphone with a frame rate of 240 Hz (HD 1080p). According to the guidelines of Bogataj et al., the smartphone was placed on a tripod and positioned approximately 1.5 m behind the participant in the frontal plane at a height of approximately 10 cm [20,38]. The My Jump Lab app uses video analysis algorithms after recording a jump to estimate key parameters, such as JH. Similar to the My Jump 2 app, jump analysis also involves marking the take-off and landing moments on the timeline to estimate JH based on the jumper’s flight time. For all CMJs, smartphone recordings and measurements on the force platform were performed simultaneously.

### 2.3. Data Analysis

The VGRF data vs. time for the 132 trials (3 trials × 22 participants × 2 LE) were exported as files with the .xlsx extension. Using Microsoft Excel 2019, the vertical displacement values were calculated based on numerical double integration of the VGRF values according to Simpson’s method. Formulas (1) and (2) were used:

(1) $$a_{z}=\frac{R_{z}}{m}-g$$

where: a_z_—vertical acceleration, m—body mass, R_z_—vertical ground reaction force, and g—gravity acceleration (9.80665 m∙s^−2^). The m results were calculated using the formula m = VGRF/g for the VGRF values measured by the force platform in the standing phase of the CMJ during body stabilization. Then, the mean m calculated from the values for the stabilization phase was substituted into Formula (1).

(2) $$d_{z}(t)=d_{oz}+\int v_{z}(t)dt$$

where: d_z_(t)—vertical displacement of the jumper’s CM vs. time, d_oz_—initial vertical displacement, and v_z_(t)—vertical velocity vs. time.

**Figure 1:**
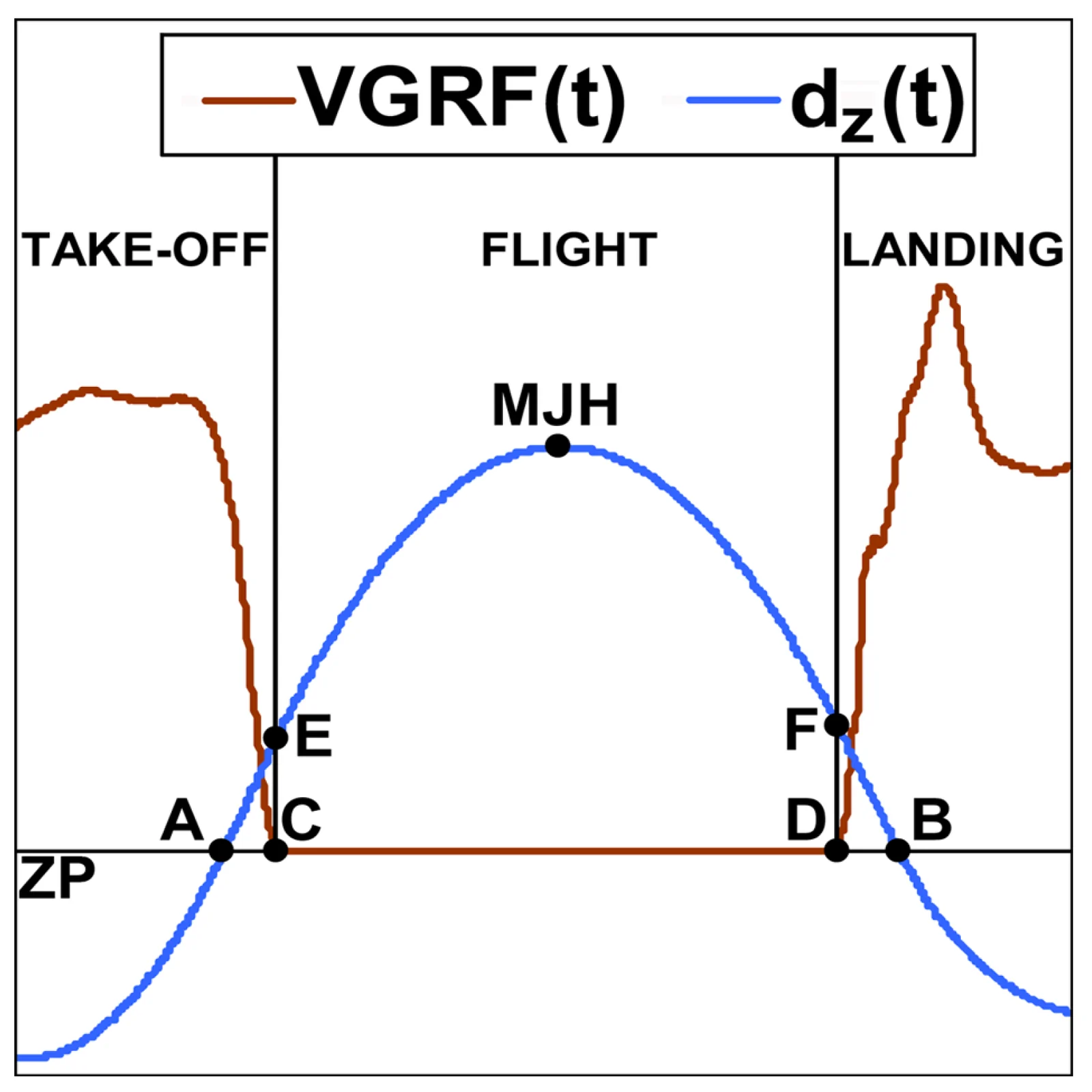
The VGRF(t) and d_z_(t) graphs for the representative trial of the countermovement jump. VGRF—the vertical ground reaction force, d_z_—the vertical displacement of the jumper’s center of mass, MJH—the maximum jump height, ZP—the vertical zero position, A—the point for dz = 0 during the take-off phase, B—the point for dz = 0 during the landing phase, C—the starting point of the jumper’s flight phase, D—the ending point of the jumper’s flight time, E—the ending point of the take-off phase, F—the starting point of the landing phase.

Considering the d_z_(t) graph, the highest value of d_z_, which is the JH of the CMJ, was determined. Figure 1 shows VGRF(t) and d_z_(t) graphs for the representative trial of the CMJ.

JH was also calculated based on the flight time (FT) of the jumper according to Bosco’s formula (3) [15].

(3) $$JH=1.22625\cdot FT^{2}$$

where: FT—time between point C and point D when VGRF = 0 (Figure 1).

In the My Jump Lab app, FT was determined as the time between the first frame of the recording when the foot of the jumper was lifted off the ground and the first frame of the recording when the first foot touched the ground during the landing.

**Figure 2:**
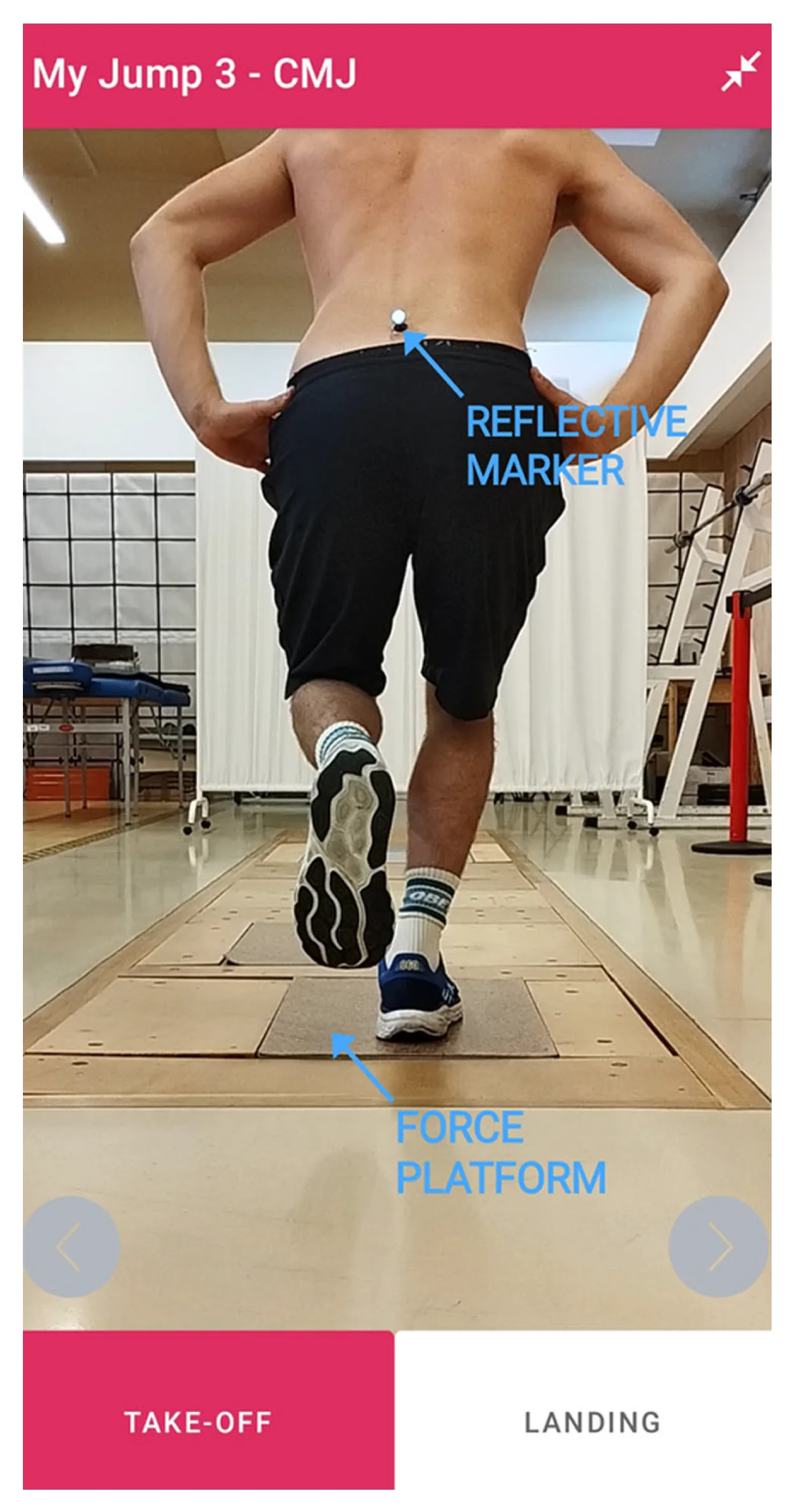
A jumper with a reflective marker while performing a single-leg countermovement jump on a force platform. The movement was recorded using the My Jump Lab app.

The My Jump Lab app was also used to estimate the JH according to the My Jump Lab-M method, i.e., based on the FT of the reflective marker placed on the jumper’s sacrum [20] (Figure 2).

Before the tests, a special spherical marker (diameter of 20 mm) reflecting infrared rays was placed on the sacrum between the posterior superior iliac spines of each jumper [20,39]. Hence, all participants performed CMJs with this marker. The analysis of recorded jumps in the My Jump Lab app began by establishing the vertical zero position (ZP) of this marker in the standing phase of the CMJ. Then, using the “draw angles” option, a thin horizontal line was drawn through the center of the marker in its ZP during the standing phase. The starting frame in the take-off phase and the ending frame in the landing phase were determined when the center of this marker was on this horizontal line. After these settings, the My Jump Lab app calculated the marker’s FT between these frames and then the JH according to Formula (3). The mean error was 0.3 cm for the JH range between 0 cm and 65 cm. All recordings and calculations using the My Jump Lab app were performed by the same researcher.

### 2.4. Statistical Analysis

Normality of distribution was determined using the Shapiro–Wilk test. Repeated measures analysis of variance (ANOVA) with two factors (method [force platform, My Jump Lab, My Jump Lab-M] × LE [D, ND]) for JH was performed. A Bonferroni correction for multiple pairwise comparisons was used. Sphericity was assessed using Mauchly’s test. The Greenhouse–Geisser adjustment was made when sphericity was violated. The effect size was determined using partial eta-squared (η^2^). According to Cohen’s guidelines, values of η^2^ were small at 0.01, medium at 0.06 and large at 0.14 [40]. To analyze the absolute agreement between the two measurement tools, an intraclass correlation coefficient (ICC) with a 95% confidence interval (CI) related to the 2-way random single measures (2,1) was used. Additionally, Bland–Altman plots showing systematic bias and the limits of agreement (LOA) for the two compared instruments were created [41]. Pearson’s product–moment correlation coefficient (r) was calculated. The r value was evaluated according to the following thresholds: <0.1 (trivial), 0.1–0.3 (small), 0.3–0.5 (moderate), 0.5–0.7 (large), 0.7–0.9 (very large) and 0.9–1.0 (almost perfect) [42]. Furthermore, a linear regression analysis was performed to assess the degree of relation between the two measurement tools. The absolute reliability of the measurement tools estimating the height jump for the 3 CMJ trials for each participant was examined using the ICC (2,1) [43], standard error of measurement (SEM) and the coefficient of variation (CV). The ICCs were interpreted based on the following scale: <0.5 (poor), 0.5–0.75 (moderate), 0.75–0.9 (good) and >0.9 (excellent) [44]. The SEM and CV were calculated using Equations (4) and (5):

(4) $$\mathrm{SEM}=SD\sqrt{1-\mathrm{ICC}}$$

where: SD—standard deviation for the average of the SDs from 3 trials,

(5) $$\mathrm{CV}=\frac{SEM}{Mean}$$

where: mean—the arithmetic average of the 3 trials. The significance level alpha was set at *p* < 0.05. The statistical analysis was performed in SPSS Statistics for Windows, version 31.0 (Armonk, NY, USA: IBM Corp).

## 3. Results

The means and standard deviations of JH are presented in Table 1. The results of Mauchly’s test were W = 0.773, *p* = 0.001 (method factor) at a Greenhouse–Geisser adjustment of 0.815, and W = 1, with *p* = 1.0 (LE factor). The analysis indicated that all data were normally distributed (*p* > 0.05). Considering the JH values, the analysis revealed (1) a significant main effect (*p* < 0.001) and a large effect size (η^2^ = 0.924 at F_2,106_ = 793.2) for the method factor and (2) a significant main effect (*p* = 0.049) and a medium effect size (η^2^ = 0.058 at F_1,65_ = 4.0) for the LE factor.

Pairwise comparisons between the measurement tools showed (1) a significant (*p* < 0.001) lower mean JH value for the My Jump Lab than the force platform, by 49.2% (ND) and by 49.4% (D), and (2) non-significant differences (below 2.0%) between the My Jump Lab-M and force platform (*p* = 1.0) for both LEs.

The results for the ICC and 95% CI are presented in Table 2. The ICCs showed poor agreement between the My Jump Lab and force platform (*p* < 0.001), as well as moderate (ND) and good (D) absolute agreement between My Jump Lab-M and the force platform (*p* < 0.001).

**Figure 3:**
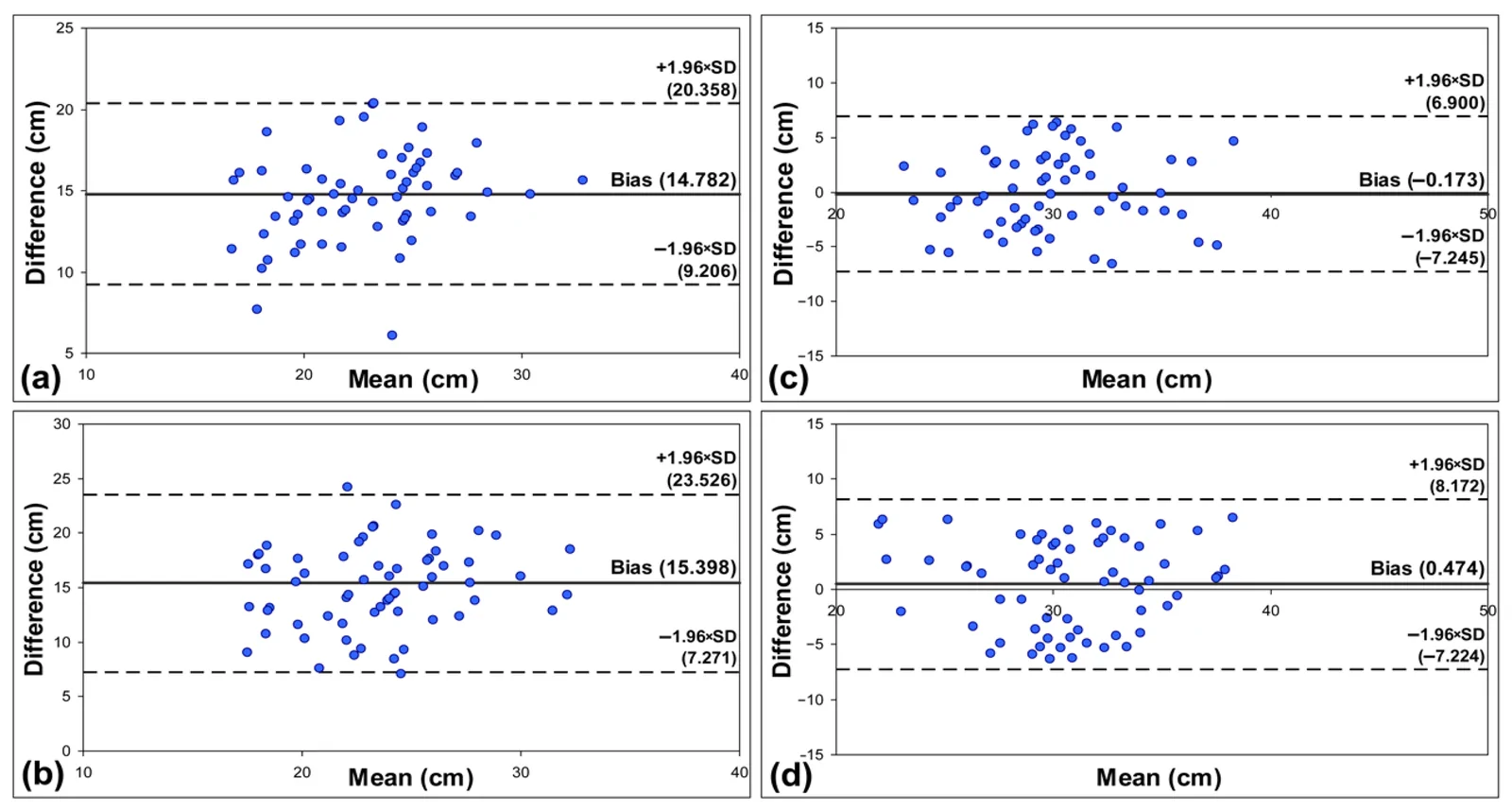
Bland–Altman plots for the following: (**a**) My Jump Lab and force platform (ND), (**b**) My Jump Lab and force platform (D), (**c**) My Jump Lab-M and force platform (ND), (**d**) My Jump Lab-M and force platform (D). My Jump Lab—includes the method of the jumper’s flight time, My Jump Lab-M—includes the method of the flight time of the marker placed on the jumper’s sacrum, ND—non-dominant lower extremity, D—dominant lower extremity, SD—standard deviation for the average difference between the two instruments, +1.96 × SD—upper limit of agreement, −1.96 × SD—lower limit of agreement, bias—average difference between the two instruments.

The Bland–Altman plots (Figure 3) showed the following bias: (1) approximately 14.8 cm at an LOA range of approximately 11.2 cm (ND) and approximately 15.4 cm at an LOA range of approximately 16.3 cm (D) for the My Jump Lab vs. force platform (Figure 3a,b), and (2) approximately –0.2 cm at an LOA range of approximately 14.1 cm (ND) and approximately 0.5 cm at an LOA range of approximately 15.4 cm (D) for the My Jump Lab-M vs. force platform (Figure 3c,d).

**Figure 4:**
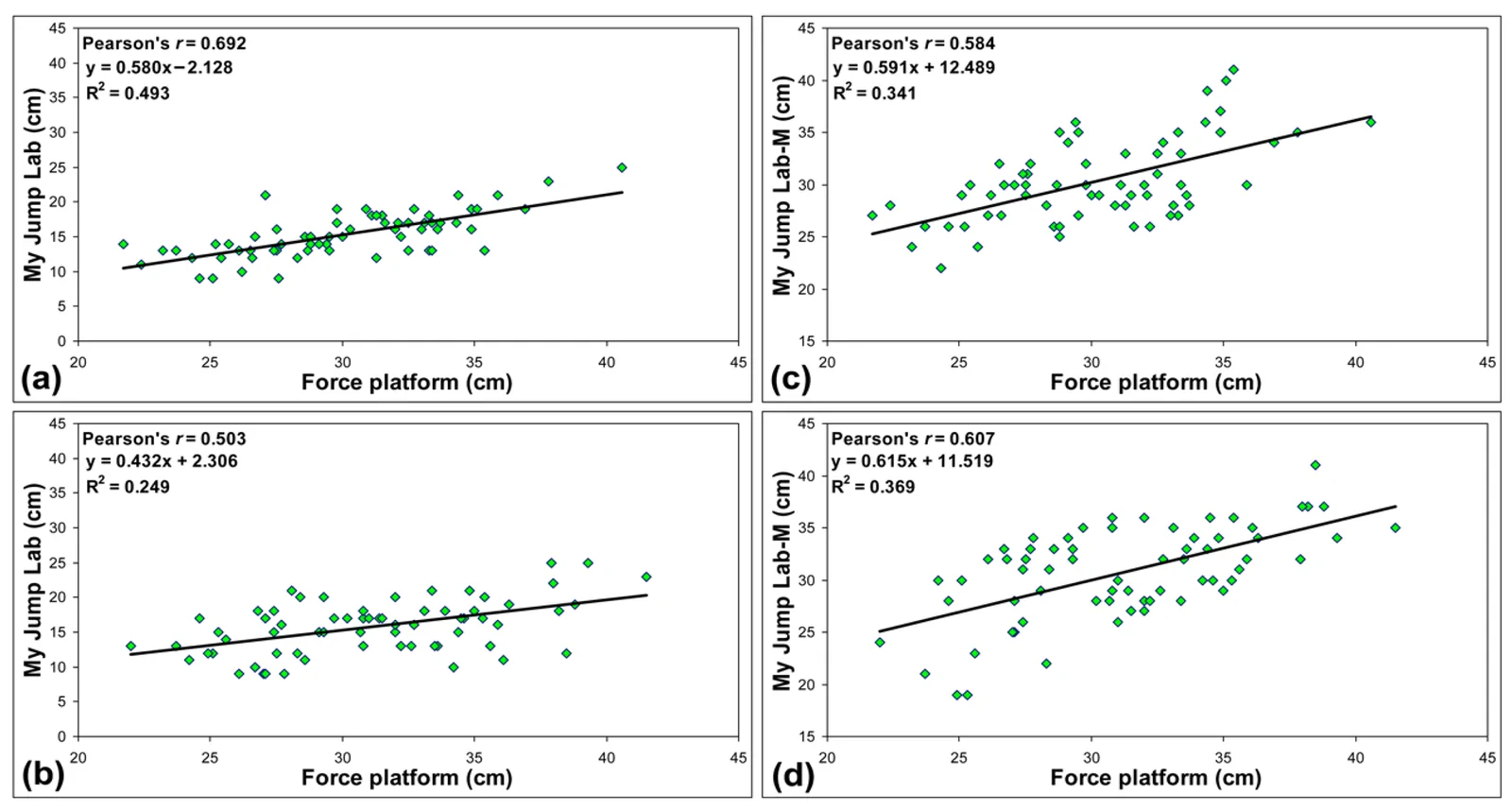
Plots of the correlation between the following: (**a**) My Jump Lab and force platform (ND), (**b**) My Jump Lab and force platform (D), (**c**) My Jump Lab-M and force platform (ND), (**d**) My Jump Lab-M and force platform (D). My Jump Lab—includes the method of the jumper’s flight time, My Jump Lab-M—includes the method of the flight time of the marker placed on the jumper’s sacrum, ND—non-dominant lower extremity, D—dominant lower extremity, r—Pearson’s product–moment correlation coefficient, x and y—variables for the linear regression, R^2^—the coefficient of determination.

Furthermore, the results for Pearson’s r indicated (1) a large (ND, D) correlation between My Jump Lab and the force platform (*p* < 0.001) (Figure 4a,b) and (2) a large (ND, D) correlation between My Jump Lab-M and the force platform (*p* < 0.001) (Figure 4c,d).

The ICC values for internal consistency (>0.8) at SEM = 0.6–1.8 and CV = 0.04–0.07 indicated good and excellent reliability for all measurement tools estimating the JH for the three CMJs performed by each participant (*p* < 0.001) (Table 3).

## 4. Discussion

In this study, JH during a single-leg CMJ was estimated using the My Jump Lab app installed on an iPhone 13 smartphone, based on the jumper’s flight time (first method) and the flight time of a reflective marker placed on each participant’s sacrum (second method). Both measurement tools were compared to the accurate method of double integration for VGRF values recorded during jumps on an AMTI force platform (gold standard). For this purpose, the absolute agreement (ICC, Bland–Altman and correlation plots) between My Jump Lab and the force platform, and between My Jump Lab-M and the force platform, was assessed. In addition, reliability was determined as the internal consistency between measurements for each of the three tools.

Comparisons between My Jump Lab-M and the force platform revealed a generally good absolute agreement. Specifically, this accuracy is reflected in the ICC (approximately 0.75), Bland–Altman plots showing a small systematic bias in the JH difference (approximately 0 cm), and all measurement points between the lower LOA and upper LOA, as well as a large correlation coefficient (approximately 0.6). This good absolute agreement can be attributed to the excessively wide ICC range for the 95% CI (approximately 0.25–0.27), ranging from a lower bound of approximately 0.58 (moderate agreement) to an upper bound of approximately 0.85 (good agreement). However, the good agreement between My Jump Lab-M and the gold standard should be considered an insufficient result for the correct assessment of jump height, as excellent validity (more than 0.9) for a given measurement tool is required. Despite this, very similar mean JH results between the two methods were found; the values of this variable were only higher by approximately 0.3% (ND) and only lower by approximately 1.6% (D) for My Jump Lab-M than for the force platform. Thus, the use of this method in the single-leg CMJ test provided similar jump height values to those obtained by the method generally considered the most accurate, i.e., using the jumper’s CM displacement trajectory as a result of the double integration of VGRF values [45,46,47,48].

In contrast, data analysis revealed poor agreement (an ICC of approximately 0.1 for ND and D) and a large systematic bias in the JH difference (approximately 15 cm) between My Jump Lab and the force platform. Furthermore, a significantly lower JH by 49.2% (ND) and by 49.4% (D) was observed for My Jump Lab compared to the jumper’s CM displacement trajectory method (force platform). The use of these two different methods of estimating JH resulted in such large differences because the CM of the jumper performing the CMJ is already at a certain height (relative to the vertical zero position) at the end of the take-off phase during plantar flexion (Figure 1, point E) and at the beginning of the landing phase during dorsiflexion (Figure 1, point F). Therefore, the jumper’s flight time (My Jump app) is shorter than the CM displacement time between its vertical zero positions during take-off and landing. Considering this the most accurate method of assessing vertical jump height (gold standard), Murawa et al. [20] also demonstrated poor absolute agreement (ICC = 0.362) between the My Jump 2 app and the AMTI force platform, as well as significantly lower JH values during bilateral CMJ for this app than for the force platform. It is worth adding that the observed difference of approximately 49% between the force platform and My Jump Lab app indicates a discrepancy that may rather be due to the different principles and mechanisms underlying the two measurement tools. Although both methods reflect the same phenomenon, they are not directly interchangeable and may yield substantially different absolute values. Therefore, the results obtained using these methods should not be compared in absolute terms or interpreted as equivalent measures.

The results for JH for the three CMJ ND trials and three CMJ D trials performed by each participant demonstrated good and excellent reliability of the measurement tools. These values indicate the high internal consistency of JH, meaning high measurement stability for this variable, estimated using the force platform and both methods associated with the My Jump Lab app. Other researchers have also reported the very good reliability of the My Jump app [16,23,49].

Additionally, the analysis revealed statistically non-significant differences in mean JH values between the D and ND for all methods, although a significant main effect was observed for the LE factor. However, the result of this effect did not translate into significant differences in the analyzed pairwise comparison of the LE factor after considering the Bonferroni correction. The non-significant inter-limb asymmetry (approximately 1–4%), calculated using the Vagenas and Hoshizaki formula [50], is as expected since the evaluation included healthy, recreationally active men who jumped biomechanically similarly on both the right LE and left LE. It is worth noting that participants achieved, on average, slightly higher CMJ height for the D than the ND. Furthermore, measurements using the force platform indicated slightly stronger (1) take-offs with the D (VGRF = 1407.1 ± 225.2 N) than with the ND (VGRF = 1367.6 ± 249.7 N) and (2) landings on the D (VGRF = 2403.7 ± 462.5 N) than on the ND (VGRF = 2384.6 ± 565.6 N). In addition to comparing JH between LEs, physiotherapists commonly assess inter-limb asymmetries of force (and power) for both take-off and landing in the vertical jump. This is a key index when monitoring athletes’ rehabilitation progress, for example, after ACL reconstruction. However, unlike the My Jump app, detecting these asymmetries is provided solely by measurements using the force platform.

This study has limitations. Firstly, test–retest reliability was not assessed due to the lack of data from the second measurement session. Various problems experienced by some participants prevented the same study group from being assembled and the CMJ tests from being repeated in the planned second session. Secondly, this study included healthy, recreationally active male students with a uniform right LE dominance. Therefore, it is difficult to relate these results to data obtained by athletes. Furthermore, these findings cannot be directly extrapolated to clinical populations, particularly patients after ACL reconstruction who have different functional and neuromuscular conditions. Thirdly, the movement of the reflective marker placed on the sacrum should not be considered a direct representation of the jumper’s CM trajectory. Changes in body configuration, segmental movements, and pelvic rotation may influence the marker’s position independently of the CM. Fourthly, a detailed analysis of inter-limb asymmetry was not performed in this study. Therefore, the potential usefulness of the evaluated method for monitoring asymmetry, especially in the context of ACL rehabilitation, should be considered a potential application requiring further verification, rather than as a direct conclusion resulting from the current study. Fifthly, women were not recruited for the study. This assumption was made to obtain a group that was as homogeneous as possible in terms of gender and related anthropometric and neuromuscular differences that may influence CMJ height. Similarly, Balsalobre et al. [16], in their first article on the validation of the My Jump app, also limited their study to men.

## 5. Conclusions

A review of the available literature suggests that this study can be considered the first to compare jump height for single-leg CMJs between the My Jump Lab app and a force platform. This study indicated that My Jump Lab-M (new method) was a more valid tool than the commonly used My Jump Lab app. Furthermore, comparisons revealed very similar JH values between the My Jump Lab-M and the gold standard. However, there was only moderate to good agreement between the My Jump Lab-M and the method of double integration of VGRF values. Therefore, this new method may not provide an objective assessment of the single-leg CMJ height. Undoubtedly, further tests are needed to assess the My Jump Lab-M method during single-leg vertical jumps in healthy and rehabilitated athletes of both genders. In summary, for objective diagnostics, physiotherapists and other practitioners should primarily rely on results obtained using the most accurate method of double integration of the VGRF.

## Figures and Tables

**Table 1:** Means and standard deviations of the variables for the measurement tools.

Variable	My Jump Lab	My Jump Lab-M	Force Platform
JH (cm), ND	15.3 ± 3.2 *	30.3 ± 4.0	30.1 ± 3.9 *
JH (cm), D	15.8 ± 3.8 *	30.7 ± 4.5	31.2 ± 4.4 *

Notes: My Jump Lab—includes the method of measuring the jumper’s flight time, My Jump Lab-M—includes the method of measuring the flight time of the marker placed on the jumper’s sacrum, JH—countermovement jump height, ND—non-dominant lower extremity, D—dominant lower extremity, *—significant difference between the force platform and My Jump Lab.

**Table 2:** The concurrent validity of the My Jump Lab app compared to the force platform for estimating the countermovement jump height.

Variable	My Jump Lab vs. Force Platform	My Jump Lab-M vs. Force Platform
ICC (–), ND	0.137	0.740
ICC (–), D	0.117	0.756
95% CI (–), ND	−0.035–0.443	0.575–0.841
95% CI (–), D	−0.061–0.387	0.602–0.850

Notes: My Jump Lab—includes the method of the jumper’s flight time, My Jump Lab-M—includes the method of the flight time of the marker placed on the jumper’s sacrum, ND—the non-dominant lower extremity, D—the dominant lower extremity, ICC—intraclass correlation coefficient, CI—confidence interval.

**Table 3:** The reliability of the measurement tools for the countermovement jump height.

Variable	My Jump Lab	My Jump Lab-M	Force Platform
ICC (–), ND	0.961	0.803	0.902
ICC (–), D	0.923	0.894	0.893
95% CI (–), ND	0.920–0.982	0.586–0.909	0.801–0.956
95% CI (–), D	0.843–0.965	0.785–0.953	0.783–0.952
SEM (–), ND	0.642	1.766	1.232
SEM (–), D	1.059	1.452	1.440
CV (–), ND	0.042	0.058	0.041
CV (–), D	0.067	0.047	0.046

Notes: My Jump Lab—includes the method of the jumper’s flight time, My Jump Lab-M—includes the method of the flight time of the marker placed on the jumper’s sacrum, ND—the non-dominant lower extremity, D—the dominant lower extremity, ICC—intraclass correlation coefficient, CI—confidence interval, SEM—standard error of measurement, CV—coefficient of variation.

## Data Availability

The data are available upon request from the corresponding author.

## Abbreviations

The following abbreviations are used in this manuscript:

CMJ	Countermovement jump
JH	Jump height
D	Dominant lower extremity
ND	Non-dominant lower extremity
CM	Center of mass
VGRF	Vertical ground reaction force
IPAQ	International Physical Activity Questionnaire
LE	Lower extremity
ZP	Vertical zero position
FT	Flight time
ICC	Intraclass correlation coefficient
CI	Confidence interval
LOA	Limit of agreement
SEM	Standard error of measurement
CV	Coefficient of variation
SD	Standard deviation
